# Dynamics of IgM and IgG Antibody Response Profile against Linear B-Cell Epitopes from Exoerythrocytic (CelTOS and TRAP) and Erythrocytic (CyRPA) Phases of *Plasmodium vivax*: Follow-Up Study

**DOI:** 10.3390/antib13030069

**Published:** 2024-08-15

**Authors:** Cinthia Magalhães Rodolphi, Isabela Ferreira Soares, Ada da Silva Matos, Rodrigo Nunes Rodrigues-da-Silva, Marcelo Urbano Ferreira, Lilian Rose Pratt-Riccio, Paulo Renato Rivas Totino, Kézia Katiani Gorza Scopel, Josué da Costa Lima-Junior

**Affiliations:** 1 Research Centre of Parasitology, Department of Parasitology, Microbiology and Immunology and Post-Graduation Program in Biological Science, Federal University of Juiz de Fora, Juiz de Fora 36036-900, Brazil; cinthiarodolphi@gmail.com; 2 Laboratory of Immunoparasitology, Oswaldo Cruz Institute, Fiocruz, Rio de Janeiro 21040-900, Brazil; isaferreirasoares@gmail.com (I.F.S.); adamatos.sm@gmail.com (A.d.S.M.); 3 Laboratory of Hantaviruses and Rickettsioses, Oswaldo Cruz Institute, Fiocruz, Rio de Janeiro 21040-900, Brazil; rodrigo.nunes@bio.fiocruz.br; 4 Department of Parasitology, Institute of Biomedical Sciences, University of São Paulo, Sao Paulo 05508-220, Brazil; muferrei@usp.br; 5 Laboratory for Malaria Research, Oswaldo Cruz Institute, Fiocruz, Rio de Janeiro 21040-900, Brazil; riccio@ioc.fiocruz.br (L.R.P.-R.); prtotino@ioc.fiocruz.br (P.R.R.T.); 6 Center for Research, Diagnosis, and Training in Malaria of Fiocruz, Rio de Janeiro 21040-900, Brazil

**Keywords:** malaria, humoral immune response, PvCelTOS, PvTRAP, PvCyRPA

## Abstract

Malaria is a serious health problem worldwide affecting mainly children and socially vulnerable people. The biological particularities of *P. vivax*, such as the ability to generate dormant liver stages, the rapid maturation of gametocytes, and the emergence of drug resistance, have contributed to difficulties in disease control. In this context, developing an effective vaccine has been considered a fundamental tool for the efficient control and/or elimination of vivax malaria. Although recombinant proteins have been the main strategy used in designing vaccine prototypes, synthetic immunogenic peptides have emerged as a viable alternative for this purpose. Considering, therefore, that in the Brazilian endemic population, little is known about the profile of the humoral immune response directed to synthetic peptides that represent different *P. vivax* proteins, the present work aimed to map the epitope-specific antibodies’ profiles to synthetic peptides representing the linear portions of the ookinete and sporozoite cell passage protein (CelTOS), thrombospondin-related adhesive protein (TRAP), and cysteine-rich protective antigen (CyRPA) proteins in the acute (AC) and convalescent phases (Conv30 and Conv180 after infection) of vivax malaria. The results showed that the studied subjects responded to all proteins for at least six months following infection. For IgM, a few individuals (3–21%) were positive during the acute phase of the disease; the highest frequencies were observed for IgG (28–57%). Regarding the subclasses, IgG2 and IgG3 stood out as the most prevalent for all peptides. During the follow-up, the stability of IgG was observed for all peptides. Only one significant positive correlation was observed between IgM and exposure time. We conclude that for all the peptides, the immunodominant epitopes are recognized in the exposed population, with similar frequency and magnitude. However, if the antibodies detected in this study are potential protectors, this needs to be investigated.

## 1. Introduction

Considered a serious global public health problem, malaria, a disease caused by protozoa of the genus *Plasmodium*, was responsible for about 249 million cases, and around 608 thousand deaths in 2022 [1]. Plasmodia are protozoa belonging to the phylum Apicomplexa, with two of the species being the main causative agents of malaria in the world, *P. falciparum* and *P. vivax* [2].

Amidst the human malaria parasites, *P. vivax* is the most widely distributed [3]. At least in part, the ability of this parasite to develop a dormant life stage in the liver of its host, called hypnozoite, causing late relapses [4,5], as well as the early circulation of its sexual stage, gametocytes, in the peripheral blood, have contributed to its maintenance in endemic areas of the globe [6]. Together with huge spatial distribution, the emergence of chemo-resistant parasites, the resistance of the vector to insecticides, and the lack of an effective vaccine are the main reasons for the search for new strategies against vivax malaria [7,8]. The RTS,S/AS01E vaccine, under the commercial name Mosquirix™, was the first human vaccine against malaria to undergo a high level of regulation, with a protection rate against severe symptoms of around 30% [9,10]. However, in 2023, a new vaccine, named the R21/Matrix-M, was also recommended by the WHO, showing a protection rate of around 66% against clinical episodes of disease [11]; both vaccines are aimed at children living in areas of high and moderate transmission and are only directed against *P. falciparum* [1,10,11]. Therefore, numerous efforts are still necessary until an effective vaccine may be accessible for all plasmodia species [12].

Among all *P. vivax* proteins, CelTOS, TRAP, and CyRPA have been widely studied and, recently, considered promising for vaccine development [13,14,15]. CelTOS is a highly conserved protein in *Plasmodium* species that is secreted in micronemes and subsequently translocated to the surface of the parasite to mediate its invasion into hepatocytes, as well as its locomotion and cell passage in both vertebrate and invertebrate hosts [16,17]. TRAP is also a highly conserved sporozoite protein, acting on parasite motility [18,19] and, together with the circumsporozoite protein (CS), is fundamental for the invasion of both the salivary glands of the insect vector and the hepatocytes of the vertebrate host [20,21]. CyRPA is found in the micronemes of merozoites, helping erythrocytes to invade through the formation of a multiprotein complex between the merozoite and the erythrocyte [22]. To date, studies show that these proteins, present in the fundamental stages of the parasite’s life cycle, are immunogenic and that antigen-specific antibodies correlate with naturally acquired immunity in exposed populations [23,24].

Most of the available studies that investigate the antibody response against malarial antigens in exposed populations use recombinant proteins [25,26,27,28]. However, despite advances in the synthesis of recombinant proteins, their use still has many problems, such as risks of contamination during production and the possibility of developing autoimmune responses [12,29]. On the other hand, peptides have become increasingly common in scientific research due to advantages such as the ability to construct, validate, and check their effectiveness in silico before experimental testing, avoiding spending money and time [30]. Previous studies suggest that linear peptides can induce immune responses associated with protection both in experimental models and in humans [31,32,33,34]. However, especially with regard to *P. vivax*, few studies have evaluated the immunogenicity and antibody response of B-cell linear epitopes from different proteins in populations naturally exposed to malaria, especially after an episode of disease [12]. Therefore, in this study, we map the antibody response profile, during acute and convalescent phases of vivax malaria, against linear peptides representing PvCelTOS, PvTRAP, and PvCyRPA proteins in exposed subjects living in low transmission regions of Brazil.

## 2. Materials and Methods

### 2.1. Study Area and Participants

The complete description of the study area and patients’ profiles can be found in detail in Soares and colleagues (2019; Table A1) [35]. Briefly, the selected study area belongs to the Brazilian Amazon region and comprises the municipalities of Nova California, in Rondônia state and, Acrelândia and Plácido de Castro, in Acre state. Venous blood was collected from 56 patients presenting symptoms of malaria. The infection was initially detected by thick blood smear and, next the infective parasite species was confirmed by PCR. After 30- and 180-days post-infection (named convalescent phase; Conv 30 and Conv 180) from 56 acute individuals, only 46 and 24 patients, respectively, were located and had a new blood sample collected. All patients signed the consent form, completed a questionnaire reporting the previous number of malaria infections and the exposure time in the endemic area, and then infected individuals were properly treated following the guidelines of the Brazilian Ministry of Health. In addition to the infected individuals, blood was collected from 28 individuals, named cohabitants (CH), who were residents of the study area without active infection for at least 1 year. Twenty individuals who were not residents of an endemic area, named as non-exposed controls (NEC), were also recruited for the study to be used in the calculation of the cut-off value. The plasma, obtained from venous blood, was stored at −20 °C until use.

The inclusion and exclusion criteria for individuals in the study are described in a previous study [35]. Namely, the initial condition for inclusion of acute participants was to present infection by *Plasmodium* sp. and not have a history of any autoimmune disease or any other type of immunosuppression (such as HIV/AIDS, hepatitis, leishmaniasis, and pregnancy). The absence of reinfection was a criterion for maintaining the individual in the study throughout the segment. The inclusion and exclusion criteria for individuals in the CH group were, in addition to health conditions, the absence of malaria infection for at least one year before blood collection, regardless of whether they had previously contracted malaria. For unexposed individuals (NEC), the main inclusion criterion was never having visited a malaria-endemic area before the study.

### 2.2. Synthetic Antigens

To determine the levels of antibodies specific to PvCelTOS, PvTRAP and PvCyRPA, the peptides PvCelTOS^(I133-G147)^, PvTRAP^(P344-G374)^ and PvCyRPA^(T289-G307)^, respectively, were chosen based on previous studies [23,24], Soares et. al., 2024 (unpublished data). These studies identified and validated different epitopes as naturally immunogenic in populations exposed to infection in the Brazilian Amazon, with the most promising ones being chosen for this study, i.e., those with the highest recognition rate and highest levels.

### 2.3. ELISA

The concentration of IgG antibodies and their subclasses and IgM were measured using the enzyme-linked immunosorbent assay (ELISA) technique, according to the protocol standardized previously [23]. Briefly, MaxiSorp 96-well plates were coated with 20 µg/mL of each antigen for IgG total and 5 µg/mL for the IgG subclasses and IgM (50 µL/well) and then incubated overnight in a humidified oven at 37 °C. Next, the plates were washed 3× with 1× phosphate buffered saline (PBS) and then blocked with 200 µL of 4% bovine serum albumin (BSA) for 1h30min at 37 °C in a humid oven. Then the 4% BSA was discarded, and the samples, diluted 1:100 in 2% BSA, were added to the plates in duplicate, which were incubated again for 2 h at 37 °C in a humid oven. Next, the plates were washed 3 times with 0.05% PBS-Tween. After monoclonal antibodies diluted in 2% BSA were added to the plate (IgG 1:1000, IgG1 1:4000, IgG2 1:2000, IgG3 1:4000, IgG4 1:4000, IgM 1:1000; Sigma, St. Louis, MO, USA), which were incubated for 1 h at 37 °C in a humid oven. Finally, the plates were washed 3 times with 0.05% PBS-Tween and once with PBS 1×. IgG total was revealed with o-phenylenediamine dihydrochloride (OPD) while subclasses and IgM were revealed with 3,3′,5,5′-tetramethylbenzidine (TMB). All reactions were stopped with H_2_SO_4_ and absorbance was determined in the ELISA reader (Bio-Tek, Winooski, VT, USA) at 490 nm. The cut-off value was determined from the average of the negative controls (subjects living in the non-endemic area) plus 3 times the standard deviation. The reactivity index (RI) was obtained by dividing the absorbance value by cut-off; individuals with RI ≥ 1 were considered to have detecting antibodies while individuals with RI < 1 were considered negative.

### 2.4. Statistical Analyses

Statistical analyses were performed using the GraphPad Prism 9.5.1 program for Windows. Differences in antibody frequencies between groups were tested by the chi-square test. The magnitude of the reactivity indexes was measured using the Kruskal–Wallis test, followed by the Mann–Whitney post-test. The follow-up analyses were measured by the Friedman test, followed by the Wilcoxon test. Correlations were tested using Spearman’s test for nonparametric data and Pearson’s test for parametric data. *p* values < 0.05 were considered statistically significant.

## 3. Results

### 3.1. Patterns of IgM and IgG Antibody Response

Naturally acquired antibodies during malaria infection are considered fundamental in controlling the clinical progression of the disease [36,37]. Thus, the first parameters evaluated in this study were the frequencies of responders and the IgM and IgG antibody reactivity profiles against peptides representing the exoerythrocytic (TRAP^(P344-G374)^ and CelTOS^(I133-G147)^), and erythrocytic stage proteins (CyRPA^(T289-G307)^) of *P. vivax*. Interestingly, the frequencies of IgM and IgG positive responses for all peptides, independent of the cycle phase, were similar among infected individuals (acute phase) and CH (*p* > 0.05). However, a higher frequency of positive responses was observed for proteins from the exoerythrocytic stage, especially for IgG when compared to IgM (*p* < 0.05).

**Figure 1:**
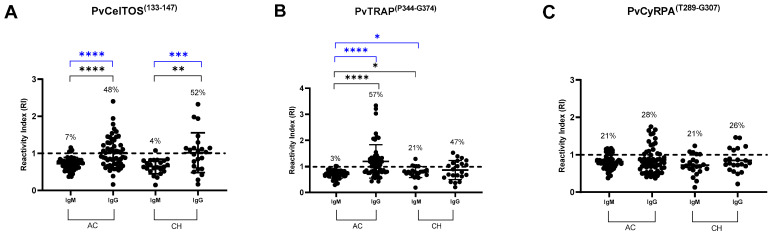
Profile of the antibody response (frequency of responder and reactivity index) against (**A**) PvCelTOS^(I133-G147)^, (**B**) PvTRAP^(P344-G374)^, and (**C**) PvCyRPA^(T289-G307)^ in the acute phase (AC) of *P. vivax* infection and cohabitant (CH) individuals. The dashed line represents the cut-off that separates responders from non-responders. The horizontal line in the scatter plots represents the median reactivity index (RI) value. Each sample was tested in duplicate by ELISA. Statistical significance of the frequency of responders (blue bars) at different points for each antigen was assessed using the chi-square test with Fischer’s post-test for multiple comparisons. Statistical significance of the RI (black bars) observed at different points for each antigen was assessed using the Kruskal–Wallis test with Mann–Whitney post-test for multiple comparisons (*p* < 0.05). Asterisks represent *p* value summary, * *p* < 0.05; ** *p* < 0.005; *** *p* < 0.0005; **** *p* < 0.0001. AC (acute), CH (cohabitants). AC: IgG and IgM: *n* = 56, CH: IgG and IgM *n* = 23.

Regarding RI, a higher range was observed for IgG. Specifically, this immunoglobulin was observed in the ranges of 1.0 to 3.3 for PvTRAP^(P344-G374)^; 1.0 to 2.4 for PvCelTOS^(I133-G147)^; and 1.0 to 1.7 for PvCyRPA^(T289-G307)^ for the individuals in the acute phase of the infection and for subjects only exposed to the infection (CH) (Figure 1A–C).

**Figure 2:**
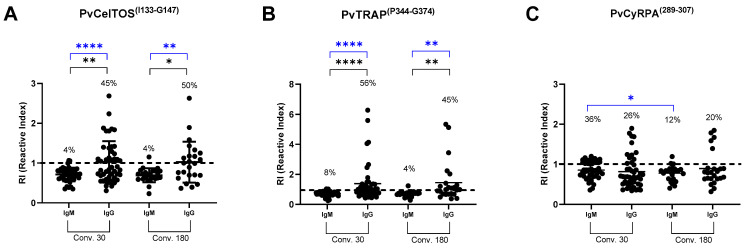
Profile of the antibody response against (**A**) PvCelTOS^(I133-G147)^, (**B**) PvTRAP^(P344-G374),^ and (**C**) PvCyRPA^(T289-G307)^ during the convalescent phase (30- and 180-days post-infection) of *P. vivax* infection. The dashed line represents the cut-off that separates responders from non-responders. The horizontal line in the scatter plots represents the median reactivity index (RI) value. Each sample was tested in duplicate by ELISA. Statistical significance of the frequency of responders (blue bars) at different points for each antigen was assessed using the chi-square test with Fischer’s post-test for multiple comparisons. Statistical significance of the RI (black bars) observed at different points for each antigen was assessed using the Kruskal–Wallis test with Mann–Whitney post-test for multiple comparisons (*p* < 0.05). Asterisks represent *p* value summary, * *p* < 0.05; ** *p* < 0.005; **** *p* < 0.0001. Conv 30 (convalescent 30 days), Conv 180 (convalescent 180 days). Conv 30: *n* = 46, Conv. 180: *n* = 24.

During the convalescent phase of the disease (30 and 180 dpi), once again a similar profile in the frequency of positive responses was observed for IgM and IgG in all peptides (Figure 2A–C); IgG being the most common immunoglobulin in the serum of the individuals studied especially for exoerythrocytic proteins. Interestingly, for PvCyRPA^(T289-G307)^ 36% of subjects presented IgM at 30dpi versus 21% observed during the acute phase of infection (Figure 1C and Figure 2C). Regarding the RI profile, the levels of IgG ranged from 1.0 to 6.2 for PvTRAP^(P344-G374)^, 1.0 to 2.6 for PvCelTOS^(I133-G147)^, and 1.0 to 1.8 for PvCyRPA^(T289-G307)^ (Figure 2A–C).

**Figure A1:**
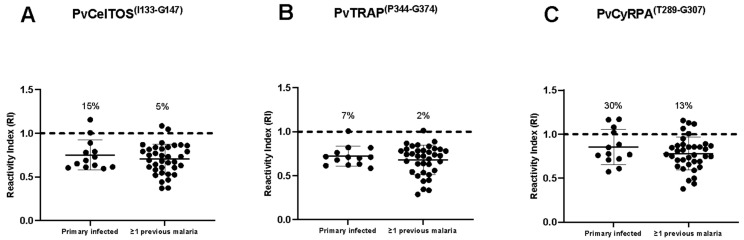
Primary infected individuals and more than 1 previous malaria individuals of (**A**) PvCelTOS^(I133-G147)^, (**B**) PvTRAP^(P344-G374)^, and (**C**) PvCyRPA^(T289-G307)^ in the acute phase of *P. vivax* for IgM. The dashed line represents the cut-off that separates responders from non-responders. The horizontal line in the scatter plots represents the median RI value. Each sample was tested in duplicate by ELISA. Statistical significance of the RI observed at different points for each antigen was assessed using the Kruskal Wallis test with Mann-Whitney post-test for multiple comparisons (*p* < 0.05). The bars represent statistical differences between the groups. Ac: *n* = 56.

We also investigated the impact of primary infection on the frequency and magnitude of IgM response for all peptides. Interestingly, the frequency of positive responders and RI were similar between individuals suffering first or multiple malarial episodes (Figure A1).

### 3.2. Pattern of Antigen-Specific IgG1, IgG2, and IgG3 Antibody Response

**Figure 3:**
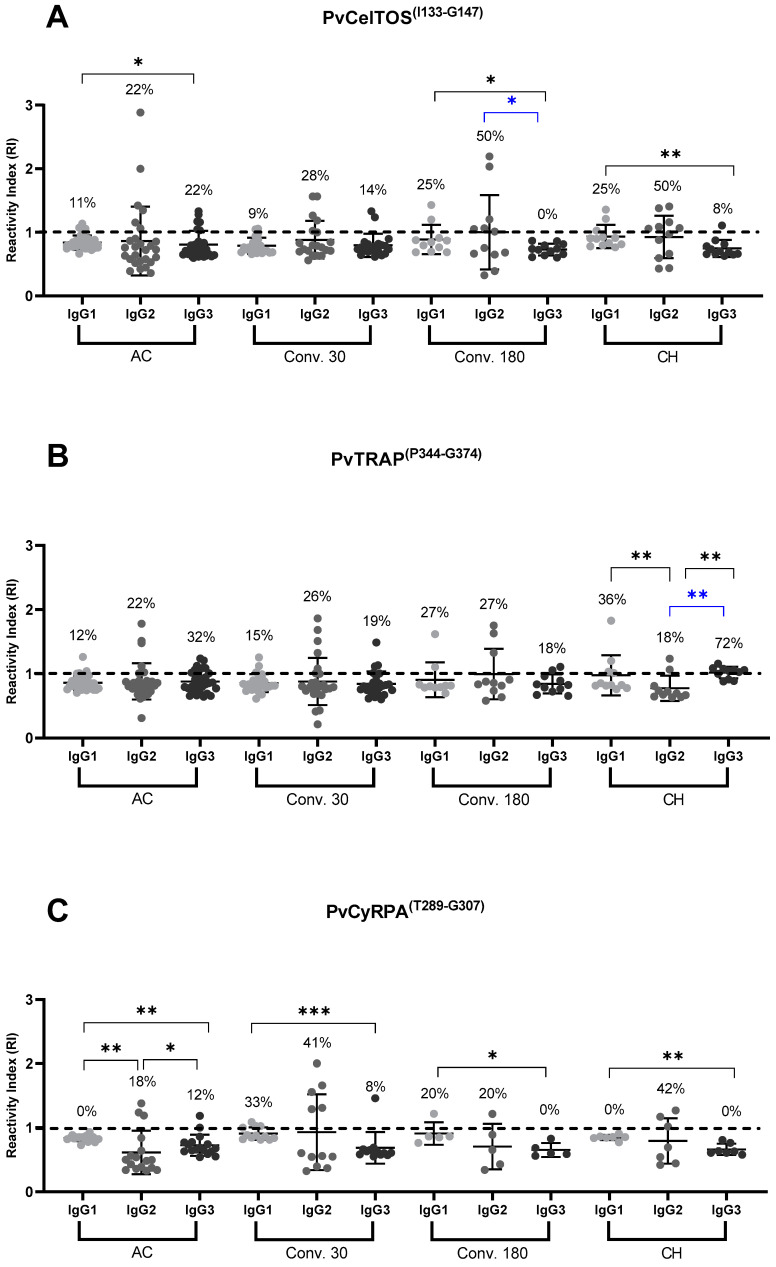
Response profile of IgG antibody subclasses (IgG1, IgG2, and IgG3) against PvCelTOS^(I133-G147)^, PvTRAP^(P344-G374)^ and PvCyRPA^(T289-G307)^ in the acute (AC) and convalescent phases of *P. vivax* (Conv 30 and Conv 180). The dashed line represents the cut-off that separates responders from non-responders. The horizontal line in the scatter plots represents the median reactivity index (RI) value. Each sample was tested in duplicate by ELISA. Statistical significance of the frequency of responders (blue bars) at different points for each antigen was assessed using the chi-square test with Fischer’s post-test for multiple comparisons. Statistical significance of the RI (black bars) observed at different points for each antigen was assessed using the Kruskal–Wallis test with Mann–Whitney post-test for multiple comparisons (*p* < 0.05). Asterisks represent *p* value summary, * *p* < 0.05; ** *p* < 0.005; *** *p* < 0.0005. 30 (convalescent 30 days), 180 (convalescent 180 days). (**A**) PvCelTOS^(I133-G147)^; acute: *n* = 27, Conv 30: *n* = 21, Conv 180: *n* = 12, CH: *n* = 12. (**B**) PvTRAP^(P344-G374)^; acute: *n* = 31, Conv 30: *n* = 26, Conv 180: *n* = 11, CH: *n* = 11. (**C**) PvCyRPA^(T289-G307)^; acute: *n* = 16, Conv 30: *n* = 12, Conv 180: *n* = 5, CH: *n* =7.

Next, individuals with IgG antibodies were selected and the frequency of responders and RI for IgG1, IgG2, and IgG3 were determined at all follow-up points. For PvCelTOS^(I133-G147)^, the highest frequency of responders was observed for IgG2, especially at 180 days of follow-up (22, 28, and 50% of responders in the acute, 30, and 180 groups, respectively). For cytophilic subclasses, IgG1 tended to increase in the frequencies of responders over time (11, 9, and 25% for acute, 30, and 180 groups, respectively). On the other hand, the frequencies of subjects who were IgG3 positive reduced over time (22, 14, and 0% of responders at the acute, 30, and 180 groups, respectively). Interestingly, the frequencies observed in the CH group were like those observed in individuals from the 180 group. Regarding RIs, these ranged from 1.0 to 2.8 (Figure 3A).

For PvTRAP^(P344-G374)^, the frequencies of positive responders and RI for all IgG subclasses were similar during follow-up (*p* > 0.05). Once again, individuals exposed to the disease, but free from infection, also presented cytophilic (IgG1 and IgG3) and non-cytophilic (IgG2) antibodies, as seen in the infected group; curiously, the highest frequency observed for IgG3 was from the CH for PvTRAP^(P344-G374)^ with 72% of responders. The RIs ranged from 1.0 to 1.8 (Figure 3B).

Finally, for PvCyRPA^(T289-G307)^, the highest frequencies of IgG subclasses were observed on day 30 post-infection. At this point of follow-up, the most prevalent subclass was IgG2 (41%), followed by IgG1 (33%) and IgG3 (8%). Interestingly, six months after infection, 20% of subjects still had detectable IgG2 antibodies. Regarding RI, the variation seen was from 1.0 to 2.0, a magnitude like those observed for the exoerythrocytic peptides (Figure 3C).

### 3.3. IgG Antibodies Longevity and Seroconversion Profile throughout Clinical Follow-Up

The seroconversion profile, as well as the longevity of the naturally acquired antibodies for all peptides were also evaluated. From the initial 56 individuals who had blood samples collected during the acute malaria and 30 days after infection, 38 individuals were relocated and had a new blood sample collected.

**Figure 4:**
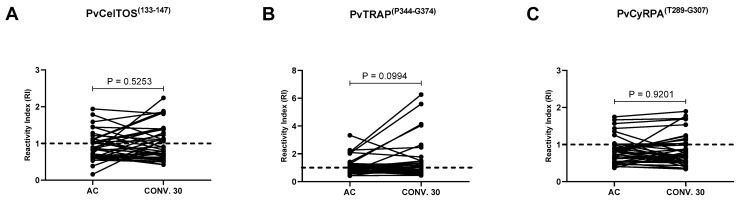
Dynamics of IgG response during acute (AC) and convalescent phase (Conv 30 dpi) of vivax malaria for (**A**) PvCelTOS^(I133-G147)^, (**B**) PvTRAP^(P344-G374)^ and (**C**) PvCyRPA^(T289-G307)^. The dashed line represents the cut-off that separates responders from non-responders. Each line with spheres at the ends represents an individual (*n* = 38). All samples were tested in duplicate by ELISA. Statistical significances were tested using the Wilcoxon test (*p* < 0.05).

For PvCelTOS^(I133-G147)^, 39% (*n* = 15) had detectable IgG antibodies during the acute phase. Of these, 67% (*n* = 10) remained positive up to day 30 of follow-up. Regarding negative individuals during the acute phase of infection (*n* = 23), only seven (30%) seroconverted until 30 days after infection (Figure 4A).

Regarding PvTRAP^(P344-G374)^, a similar profile of longevity and seroconversion was observed. Specifically, of the 19 (*n* = 38) individuals who had IgG antibodies detected during the acute phase of infection, 79% (*n* = 15) remained positive for up to 30 days. On the other hand, of individuals without detectable anti-PvTRAP^(P344-G374)^ antibodies in the acute phase of infection, only 36% (*n* = 7) seroconverted until day 30. Interestingly, for this peptide, the highest RIs were detected at point 30 of the study (Figure 4B).

Of the 38 individuals, whose antibody profile against the three selected peptides was evaluated during acute malaria and on the 30th day of convalescence, the lowest positivity rate was observed for PvCyRPA^(T289-G307)^. In this case, only 9 (23%) of the 38 individuals presented anti-PvCyRPA ^(T289-G307)^ IgG in the acute phase of infection, with 4 of them (44%) being negative by the 30th day. Regarding the seroconversion profile, it was observed that of the 29 individuals without antibodies against PvCyRPA^(T289-G307)^ in the acute phase of the disease, only 6 (20%) seroconverted until day 30 (Figure 4C).

**Figure 5:**
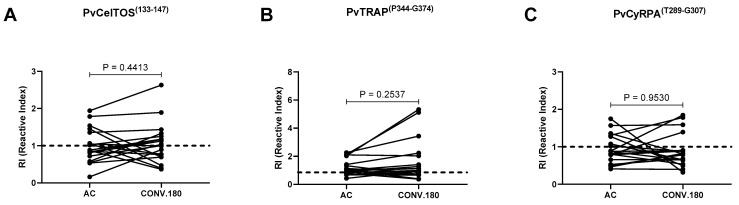
Dynamics of IgG response during acute (AC)and convalescent phase (Conv 180 dpi) of vivax malaria for (**A**) PvCelTOS^(I133-G147)^, (**B**) PvTRAP^(P344-G374)^ and (**C**) PvCyRPA^(T289-G307)^. The dashed line represents the cut-off that separates responders from non-responders. Each line with spheres at the ends represents an individual (*n* = 19). All samples were tested in duplicate by ELISA. Statistical significances were tested using the Wilcoxon test (*p* < 0.05).

Next, 19 individuals who had serum samples collected during the clinical episode of malaria and ≥180 days after infection were studied for the longevity of antibodies and seroconversion profile against the proteins of interest. From the data, it was observed that the frequency of responders in the acute phase of infection was similar for all proteins (47%, 52%, and 37% for PvCelTOS^(I133-G147)^, PvTRAP^(P344-G374)^ and PvCyRPA^(T289-G307)^, respectively). However, the highest frequency of negativity was observed for PvCyRPA^(T289-G307)^ (71% or 5/7) followed by PvCelTOS^(I133-G147)^ (56% or 5/9) and PvTRAP^(P344-G374)^ (30% or 3/10). Similar seroconversion rate was observed for PvCyRPA^(T289-G307)^ (16% or 2/12), and PvTRAP^(P344-G374)^ (22% or 2/9). The highest frequency of seroconversion was observed for PvCelTOS^(I133-G147)^ (60% or 6/10) (Figure 5).

**Figure 6:**
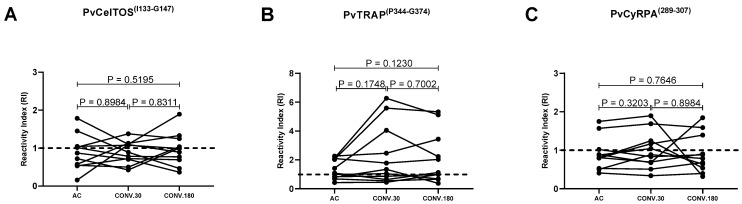
Dynamics of IgG response during acute (AC) and convalescent phase (Conv 30 and Conv 180 dpi) of vivax malaria for (**A**) PvCelTOS^(I133-G147)^, (**B**) PvTRAP^(P344-G374)^ and (**C**) PvCyRPA^(T289-G307)^. The dashed line represents the cut-off that separates responders from non-responders. Each line with spheres at the ends represents an individual (*n* = 11). All samples were tested in duplicate by ELISA. Statistical significances were tested using the Wilcoxon test.

Finally, 11 individuals with serum samples collected at all points of the follow-up were evaluated for longevity and antigen-specific antibody seroconversion (Figure 6). For PvCelTOS^(I133-G147)^, from five positive individuals at the acute phase, 60% (*n* = 3) became negative at the end of the follow-up. Seroconversion was observed in three of the six individuals who did not detect antibodies in the acute phase, 50% (*n* = 3) seroconverted and 40% (*n* = 2) remained stable (Figure 6A).

For PvTRAP^(P344-G374)^ none of the IgG-positive individuals during acute malaria became negative during follow-up. At the same time, only one seroconversion was observed during follow-up which later became negative. The magnitude of antibody response was similar during the 30- and 180-day convalescent phase (Figure 6B).

Lastly, for PvCyRPA^(T289-G307)^, from the 27% (*n* = 3) of individuals who were positive at the acute phase, 66% (*n* = 2) became negative at the end of the follow-up, only 25% (*n* = 2/8) seroconverted (Figure 6C).

### 3.4. Correlation of IgM and IgG Antibodies with Epidemiological and Immunological Parameters

We also analyzed the correlation in the patients’ RI for both IgM and IgG, with the number of previous malarial episodes, parasitemia, and time of exposure of the individual in an endemic area. As described below, the magnitude of antibody response for IgM or IgG appears not to be influenced by any of the parameters analyzed, independent of antigen (Table 1). The only correlation observed occurred between IgM and exposure time to malaria (Table 1).

### 3.5. Correlation between IgG and IgM Antibody Responses

**Figure 7:**
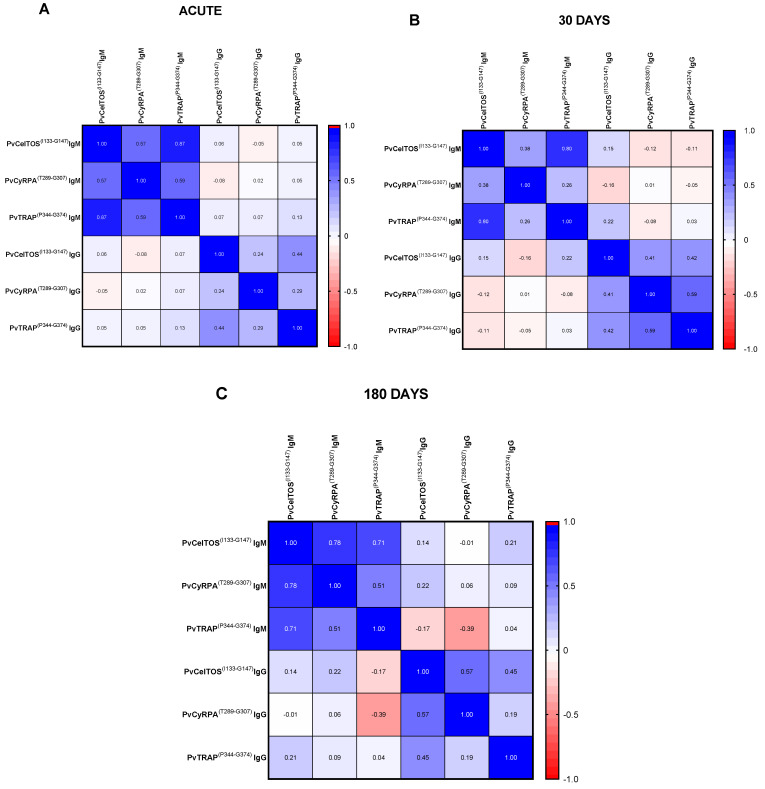
Correlation between the RIs of IgM and IgG during (**A**) acute (AC) and convalescent phases ((**B**) Conv 30 and (**C**) Conv 180) phases of infection for PvCelTOS^(I133-G147)^, PvTRAP^(P344-G374)^ and PvCyRPA^(T289-G307)^. All samples were tested in duplicate by ELISA. Statistical significances were tested by the Spearman correlation.

Last, we focused on exploring the correlation dynamics between RIs of IgM and IgG against all studied antigens, across distinct phases of our follow-up (acute phase, 30 days after infection, and 180 days). During the acute phase, we observed robust correlations among all IgM RIs, while the IgG RIs were only correlated between the pre-erythrocytic epitopes PvTRAP and PvCelTOS (r = 0.440; *p* = 0.001). (Figure 7A). However, thirty days post-infection, we noted positive yet weak correlations within IgM RIs against PvCyRPA^(T289-G307)^ and PvCelTOS^(I133-G147)^ and a strong correlation between the pre-erythrocytic antigens (r = 0.38; *p* = 0.010). This profile contrasts with IgG RIs, which, at this time point, exhibited stronger positive correlations among all antigens (*p* < 0,05). Notably, these patterns diverged from those observed in the acute phase. Moreover, interactions between IgM and IgG revealed poor negative correlations, particularly prominent between peptides originating from the exoerythrocytic phase and PvCyRPA^(T289-G307)^ from the erythrocytic phase (r = −0.165; *p* = 0.274) (Figure 7B). Finally, the 180-day phase after infection revealed that a similar correlation trend persisted between IgM and IgG RIs, in spite of sharp correlations between PvCyRPA^(T289-G307)^ and exoerythrocytic peptides for IgM (r = 0.223; *p* = 0.296) contrasted with weakened correlations between the same peptide and exoerythrocytic counterparts. Notably, a negative correlation (r = −0.394; *p* = 0.057) emerged between IgG anti-PvCyRPA^(T289-G307)^ and IgM anti-PvTRAP^(P344-G374)^ (Figure 7C).

## 4. Discussion

*P. vivax* is the *Plasmodium* species with the biggest global distribution outside Sub-Saharan Africa [38], causing alarming socioeconomic losses [39,40]. Although the scientific community’s interest in *P. vivax* infection is currently greater compared to what was seen decades ago, most malaria studies are still focused on understanding falciparum malaria, the most lethal species in the world [41,42]. At least in part, the current interest of the scientific community in understanding vivax malaria, until then considered non-lethal, is due to recent reports of severe cases and deaths associated with the infection, and the difficulties in controlling *P. vivax* compared to other species of human plasmodia [42,43,44], along with treatment failures observed around the world [45]. Although the development of an effective vaccine against *P. vivax* is considered an important strategy for combating this species, the difficulty of its in vitro culture has been considered an important obstacle to the advancement of research [46]. Another important consideration about *P. vivax* is that just a few studies, performed in countries of the African continent, Thailand, and Brazil, address the half-life of antibodies, an important parameter for choosing vaccine candidates [47,48,49,50]. Therefore, our work aimed to evaluate the antibody response, at the epitope level, in individuals exposed to malaria infection during the acute and convalescent phases of *P. vivax* infection. To our knowledge, this is the first study where the dynamics of IgM and IgG antibodies and their subclasses against linear peptides of PvCelTOS^(I133-G147)^, PvTRAP^(P344-G374),^ and PvCYRPA^(T289-G307)^ were analyzed up to six months after infection.

IgM is the first antibody to be induced during an infection, being quickly replaced by IgG after a few weeks during the immunological memory phase. As IgM is the main antibody class produced at the first contact with a new pathogen, it then presents decreased levels during reinfections with homolog parasites and, in this case, the pathogen is then eliminated by IgG produced by immunological memory cells [51]. Although it was not associated with malaria protection for a long time, it has recently gained attention for showing evidence of its participation in reducing the risk of clinical disease in children [51,52,53]. In this study, most participants (around 64%) had already undergone previous episodes of malaria, but the primary infections did not impact the IgM response profile. The low frequencies of responders and low RI for IgM may be explained by a possible production of long-lasting IgM or even the production of memory IgM, as suggested in previous studies [54,55]. However, more studies are necessary to confirm the true role of IgM in individuals with more than one previous malaria case.

IgG antibody positivity was more significant in exoerythrocytic phase peptides, with the highest levels being observed for PvTRAP^(P344-G374)^. This frequency, especially among individuals in the acute phase (57%), is higher than the findings of Matos et. al. (2019) who, when researching anti-PvTRAP^(P344-G374)^ antibodies in individuals in the acute phase and residents of an endemic area in Acre, noticed that only 32% of subjects responded to this peptide, a difference of more than 25%. The high levels of RI also drew attention in this study; Matos et. al. (2019) studied the recombinant PvTRAP protein and obtained RIs ranging from 1.0 to 4.2 [23]. Here, we observed RIs for PvTRAP^(P344-G374)^ ranging from 1.0 to 6.2. PvTRAP^(P344-G374)^ is a peptide derived from the full-length PvTRAP protein and the high rates of reactivity observed here, in comparison to the findings of Matos et. al. (2019), can be explained by PvTRAP^(P344-G374)^ being a fragment of 30 amino acids of an entire protein and therefore this fragment may be responsible for concentrating the highest antibody response compared to other peptides derived from the same recombinant protein.

PvCelTOS^(I133-G147)^ did not demonstrate results as significant as PvTRAP^(P344-G374)^, but antibodies against this peptide were also observed. In the study by Rodrigues-da-Silva and collaborators (2017), 18% of individuals living in an endemic area for malaria in Amazonas responded to the PvCelTOS recombinant protein, a frequency considered low. However, when this population was tested for the peptide PvCelTOS^(I133-G147)^, this frequency increased to 92%. However, our study shows that 48% of individuals in the acute phase and 52% of CH responded to PvCelTOS^(I133-G147)^. Regarding the RI, Rodrigues-da-Silva et.al. (2017) showed rates of responding individuals varying from 1.0 to approximately 3.5 [24], values close to those found here for individuals in the acute phase and CH (RI ranging from 1.0 to 2.4 and 2.3, respectively). Interestingly, the frequencies of responders observed for the peptides of PvCelTOS and PvTRAP (48 and 57% respectively) in our study are similar to those observed for the linear peptide representing the *P. vivax* circumsporozoite protein (PvCS; 62%) [56] and higher than the percentages observed for the VK210, VK247 and P. vivax-like peptides (34.2%, 24% and 31.5%, respectively) [57]; both studies conducted with individuals exposed to malaria in the Brazilian Amazon. It is worth emphasizing that the CS protein is one of the most important exoerythrocytic phase proteins of plasmodia and is present in the two vaccines against *P. falciparum* licensed to date.

Regarding the IgG subclasses, it is known that IgG1 and IgG3, considered cytophilic [58], are normally the most abundant in individuals who have achieved a protective status against the clinical manifestations of the disease [59,60]. IgG1 is considered the most abundant subclass [61], being stimulated by T cells and strongly binding to the Fcγ portion, mediating the activation of phagocytes and complement fixation [62], and together with IgG3, it is responsible for opsonic phagocytosis, and neutrophil-mediated death, inducing protection against clinical disease [63]. IgG3 also stands out for its high affinity for the Fcγ receptor, even greater than that of IgG1, on the surface of monocytes [64]; on the other hand, its affinity for antigens is low [65]. In addition, it has a short half-life of approximately 7 days, compared to the other subclasses [66]. Furthermore, it has been suggested that IgG2, a non-cytophilic antibody, may act as a protective antibody in in vitro functional invasion assays [67,68]. In this study, when the data on IgG subclasses were compared with the data from Matos et. al. (2019), differences were noted between the response profile of the recombinant protein PvTRAP and the peptide PvTRAP^(P344-G374)^. Specifically, while IgG1 was the subclass with the highest RI for both antigen types, the frequency of individuals responding to the recombinant protein in the acute phase of the disease (68%) [23] was significantly higher than that observed responding to peptide PvTRAP^(P344-G374)^ (13%). However, in the acute phase for PvTRAP^(P344-G374)^, IgG3 was the most predominant subclass, with 32% of positive responders. A similar profile of PvTRAP comparisons was observed for PvCelTOS^(I133-G147)^ when compared with the recombinant protein PvCelTOS. While the recombinant protein showed 66% of responders to IgG1 in its population, the peptide represented here showed 11% of responders in the acute phase and 25% of responders in the CH group [24]. The greater response of IgG1 to IgG3 in some peptides can be explained by the longer lifetime that IgG1 has, compared to IgG3. A table summarizing the main findings of this article in comparison with articles from our research group can be found in the Table A2.

The most common method to evaluate naturally acquired or vaccine-induced immunity is to measure the level of antibody response of the individual naturally exposed to the infection or who has malaria at the beginning of the infection and during a segment period, through which various responses against malaria antigens have been observed and associated with protection [69,70,71]. However, few studies around the world have investigated through long clinical follow-up, the naturally acquired immune responses using peptides from vaccine candidate proteins. In Brazil, for example, just one study developed by our group demonstrated that naturally acquired antibodies, as well as specific-antigen MBCs (memory B cells), could be detectable until at least six months after a malaria clinical episode in rural Amazonians exposed to low levels of vivax malaria transmission [47]. Similar results were obtained in this study by the analysis of the half-life and seroconversion of IgG antibody recognizing PvCelTOS^(I133-G147)^, PvTRAP^(P344-G374)^ and PvCyRPA^(T289-G307)^ during the follow-up of 30 and >180 days. For the PvTRAP^(P344-G374)^ peptide, 36% and 22% of seroconversion were observed in the segment AC-Conv30 and AC-Conv180, respectively. During a study with recombinant PvTRAP, Kosuwin et. al. (2018) analyzed the IgG antibody profile in 22 patients on days 0, 7, and 21 after infection. It was observed that six patients had antibodies against domain II of PvTRAP on day 0, and 12 had antibodies to domain IV; on D7, there was 50% seroconversion to domain II and 62.5% to domain IV; on D21, 6 of the 14 individuals who were negative on D0 became positive on domain II, while all individuals who were negative on domain IV became positive [72]. Despite this, we can highlight that regardless of seroconversion rates, the antibody response remains stable up to 180 days after infection.

Among the peptides analyzed here, PvCyRPA^(T289-G307)^ showed the lowest frequencies, which is not a surprise since França and collaborators (2017), in the study in which they identified recombinant *P. vivax* proteins for the development of vaccines, showed that PvCyRPA, despite the low frequencies of antibodies, were capable of inducing protection [15,73]. The response pattern to this antigen observed here is like that seen in the aforementioned study. The population studied here was also analyzed in the study conducted by Soares and collaborators (2019), where they also followed this population at days 0, 60, and 180 after the infection as well as the population of exposed individuals, to investigate the antibody response against two peptides from *P. vivax* erythrocytic phase: PvAMA-1^(S290–K307)^ and PvMSP9^(E795-A808)^. The authors observed that for both PvAMA-1^(S290–K307)^ and PvMSP9^(E795-A808)^, the highest frequency of responders was observed 180 days after the infection, but the highest RI was observed in the acute phase [47]. In this study, although the analysis was also in the same population studied by Soares and collaborators (2019), when we compared the erythrocytic phase proteins presented in the mentioned study and PvCyRPA^(T289-G307)^, also from the erythrocytic phase, we did not notice the same pattern. PvCyRPA^(T289-G307)^ here presents the highest frequency in the acute phase and the highest reactivity index on D30, and, in addition, the exposed group here, called the cohabitants, presented higher RIs than those seen on D30, something that was not seen in the mentioned study. Soares and collaborators (2019) also analyzed the segment on day 0 and day 30 to observe the longevity of the antibody response to the two peptides: 25% seroconversion to PvAMA-1^(S290–K307)^ and 40% seroconversion to PvMSP9^(E795-A808)^ were reported with no significant change being observed, which proves the longevity of IgG 180 after infection [47].

Calculating correlations between immune response and epidemiological parameters is important to assess whether these factors can influence the dynamics of the disease in the populations studied and how this influence occurs. Here, for almost all correlations, we did not observe significance between the response of IgG and IgM to the evaluated peptides and the exposure parameters (parasitemia, exposure time in endemic areas, and the number of previous malarial episodes). The only significant correlation observed was between PvCelTOS^(I133-G147)^ and time since last exposure for IgM (r = 0.3035; *p* = 0.0304). Rodrigues-da-Silva and collaborators (2017), in their study with the recombinant protein PvCelTOS, observed a positive correlation with the number of previous malarial episodes (r = 0.227; *p* = 0.047); here, we observed no significant correlation with the same parameter when we compared the PvCelTOS^(I133-G147)^ fragment (r = 0.1416; *p*= 0.3317) [24]. For Matos and collaborators (2019), no significant correlation was seen between the number of previous malarial episodes and the response to the recombinant protein PvTRAP (r = 0.013; *p* = 0.0832); for the exposure time parameter, a weak but significant positive correlation was observed (r = 0.194; *p* = 0.001) [23].

A great part of the differences presented here in comparison with the data presented in the literature can be explained by the differences between peptide and recombinant protein, like the low availability of epitopes present in the peptides for immunological recognition [74]. The differences observed between peptides of the same phase of the cycle may be explained by the different localizations of the proteins in the parasite; PvAMA and PvMSP9 are surface proteins while PvCyRPA is a microneme protein. Furthermore, differences in study areas, population size, collection dates, and follow-up periods must also be considered. The most important limitation in this study was the loss of patients throughout the follow-up, especially due to the impossibility of locating them. However, it is worth highlighting that data on population segments are rare, particularly in Brazil, and the data presented here for the convalescent phases of the disease related to the peptides PvTRAP^(P344-G374)^, PvCelTOS^(I133-G147)^ and PvCyRPA^(T289-G307)^ are novel and can help in understanding the dynamics of the production and maintenance of antibodies to these proteins in individuals living in the endemic area.

## 5. Conclusions

Overall, the data show that all the peptides studied were recognized, especially by the IgG antibody, which remained stable throughout the 6 months of follow-up in the absence of reinfection. Regarding the subclasses, IgG2, together with IgG3, was the most prevalent for all proteins. However, it remains to be further clarified whether the antibodies detected in this and other studies against specific peptides are capable of mediating protection.

## Figures and Tables

**Table 1:** Correlation between the RI of the acute phase for PvCelTOS^(I133-G147)^, PvTRAP^(P344-G374)^, and PvCyRPA^(T289-G307)^, with the number of previous malarial episodes, malaria exposure time (years), and parasitemia. All samples were tested in duplicate. Statistical differences were calculated using Spearman’s correlation for non-parametric data and Pearson’s correlation for parametric data.

Peptides *versus* Number of Previous Malarial Episodes	IgG	IgM
PvTRAP^(P344-G374)^	r = −0.05759; ***p* = 0.6943**	r = −0.1642; ***p* = 0.2595**
		
PvCelTOS^(I133-G147)^	r = 0.1416; ***p* = 0.3317**	r = −0.1794; ***p* = 0.2175**
		
PvCyRPA^(T289-G307)^	r = 0.06536; ***p* = 0.6555**	r = −0.1372; ***p* = 0.3471**
**Peptides *versus* Malaria Exposure Time (years)**	**IgG**	**IgM**
PvTRAP^(P344-G374)^	r = −0.07877; ***p* = 0.5827**	r = −0.2366; ***p* = 0.0945**
		
PvCelTOS^(I133-G147)^	r = 0.05917; ***p* = 0.6800**	r = −0.3035; ***p* = 0.0304**
		
PvCyRPA^(T289-G307)^	r = 0.02747; ***p* = 0.8482**	r = −0.1063; ***p* = 0.4624**
**Peptides *versus* Parasitemia**	**IgG**	**IgM**
PvTRAP^(P344-G374)^	r = 0.1972; ***p* = 0.1451**	r = 0.2383; ***p* = 0.0769**
		
PvCelTOS^(I133-G147)^	r = 0.1428; ***p* = 0.2936**	r = 0.1873; ***p* = 0.1669**
		
PvCyRPA^(T289-G307)^	r = 0.08225; ***p* = 0.5467**	r = 0.06860; ***p* = 0.6154**

## Data Availability

All data generated or analyzed during this study are included in this article.

## Appendix A

**Table A1:** Patients’ profiles. Data for continuous variables are reported as medians and interquartile ranges and compared with Kruskal–Wallis (three groups) or Mann–Whitney tests (two groups). The absolute number and proportion of males are compared with a chi-squared test.

Characteristics	NEC (*n* = 20)	CH (*n* = 23)	AC (*n* = 56)	*p*-Value
Age (y), median (IQR)	26.5 (23.0–31.0)	40.0 (23.0–60.0)	34.5 (7.0–64.0)	0.002
Number (%) of males	2 (10%)	14 (60.87%)	41 (73.22%)	<0.0001
Years of exposure to malaria	NA	35.4 (8.0–60.0)	29.5 (7.0–60.0)	0.021
Number of previous laboratory-confirmed malarial episodes, mean (SD)	NA	2.1 (3.4)	3.8 (5.4)	0.31
Time from last malarial episode, median (IQR)	NA	6 (0–30.0)	11.5 (0–20.0)	0.74

**Table A2:** Comparative profile of IgG antibody responses (% and RI) observed against the same *P. vivax* peptides in Brazilian endemic population.

Topic	Rodolphi et al.	Previous Studies
Frequency of IgG against PvTRAP^(P344-G374)^	57%	32%
RI for PvTRAP^(P344-G374)^	1–6.2	1–4.2
Frequency of IgG against PvCelTOS^(I133-G147)^	52%	92%
RI for PvCelTOS^(I133-G147)^	1–2.4	1–3.5
Subclasses for PvTRAP^(P344-G374)^	IgG3 as the prominent subclass (32%)	IgG1 as the prominent subclass (68%)
Subclasses for PvCelTOS^(I133-G147)^	IgG3 as the prominent subclass (22%)	IgG1 as the prominent subclass (66%)
